# Sex-based differences in remote monitoring of biometric, psychometric and biomarker indices in stable ischemic heart disease

**DOI:** 10.1186/s13293-022-00423-5

**Published:** 2022-04-11

**Authors:** Lili Barsky, William Speier, Garth Fuller, Susan Cheng, Andy Kim, Sandy Joung, Corey Arnold, Shivani Dhawan, Mayra Lopez, Mitra Mastali, Irene van den Broek, Janet Wei, Brennan Spiegel, Jennifer E. Van Eyk, C. Noel Bairey Merz, Chrisandra Shufelt

**Affiliations:** 1 Barbra Streisand Women’s Heart Center, Smidt Heart Institute, Cedars-Sinai Medical Center, 127 S. San Vicente Blvd, Suite A3206, Los Angeles, CA 90048 USA; 2 Medical Imaging and Informatics Group, University of California, Los Angeles, CA USA; 3 Cedars-Sinai Center for Outcomes Research and Education (CS-CORE), Los Angeles, CA USA; 4 Cedars-Sinai Medical Center, Advanced Clinical Biosystems Research Institute, Cedars-Sinai Smidt Heart Institute, Los Angeles, CA USA

**Keywords:** Precision medicine, Patient reported outcome measures, Myocardial ischemia, Biomarkers

## Abstract

**Background:**

Sex-based differences are crucial to consider in the formulation of a personalized treatment plan. We evaluated sex-based differences in adherence and remotely monitored biometric, psychometric, and biomarker data among patients with stable ischemic heart disease (IHD).

**Methods:**

The Prediction, Risk, and Evaluation of Major Adverse Cardiac Events (PRE–MACE) study evaluated patients with stable IHD over a 12-week period. We collected biometric and sleep data using remote patient monitoring via FitBit and psychometric data from Patient-Reported Outcomes Measurement Information System (PROMIS), Kansas City Cardiomyopathy (KCC) and Seattle Angina Questionnaire-7 (SAQ-7) questionnaires. Serum biomarker levels were collected at the baseline visit. We explored sex-based differences in demographics, adherence to study protocols, biometric data, sleep, psychometric data, and biomarker levels.

**Results:**

There were 198 patients enrolled, with mean age 65.5 ± 11 years (± Standard deviation, SD), and 60% were females. Females were less adherent to weekly collection of PROMIS, KCC and SAQ-7 physical limitations questionnaires (all *p* < 0.05), compared to males. There was no difference in biometric physical activity. There was a statistically significant (*p* < 0.05) difference in sleep duration between sexes, with females sleeping 6 min longer. However, females reported higher PROMIS sleep disturbance scores (*p* < 0.001) and poorer psychometric scores overall (*p* < 0.05). A higher proportion of males had clinically significant elevations of median N-terminal pro-brain natriuretic peptide (*p* = 0.005) and high-sensitivity cardiac troponin levels (*p* < 0.001) compared to females.

**Conclusions:**

Among females and males with stable IHD, there are sex-based differences in remote monitoring behavior and data. Females are less adherent to psychometric data collection and report poorer psychometric and sleep quality scores than males. Elevated levels of biomarkers for MACE are more common in males. These findings may improve sex-specific understanding of IHD using remote patient monitoring.

## Highlights

- Among patients with stable ischemic heart disease, adherence to remote psychometric data collection was higher among males than females
- There was a statistically significant (*p*<0.05) difference in sleep duration between sexes, with females sleeping 6 minutes longer. However, females reported poorer sleep quality scores.
- While females reported worse psychometric scores, there was no significant difference in remotely measured physical activity between females and males.
- Elevated levels of biomarkers for major adverse cardiac events were more often found in males than females.

## Background

Historically, medical care plan formulation was based on a patient’s report of symptoms, diagnostic testing and the clinician assimilating this data with evidence from the medical literature. However, the contemporary medical community recognizes that individuals with the same symptoms and disease often do not have the same underlying pathophenotype. Precision medicine is an evolving discipline that emphasizes the use of novel diagnostic methods, to acquire a more comprehensive understanding of an individual patient’s health beyond the hospital walls, and to develop a more targeted and individualized care plan, accordingly. While rooted in genomics and proteomics, the advent of digital health technology has significantly added to the diversity and utility of data available [1]. Indeed, the emerging capacity for remote patient monitoring has facilitated the acquisition of biometric, psychometric, patient-reported outcomes that are significant to formulating an effective precision-based approach to patient care.

Ischemic heart disease (IHD), characterized by inadequate blood flow to the myocardium, is the leading cause of death in the United States [2]. Complications may include myocardial infarction, ischemic cardiomyopathy or sudden cardiac death. IHD is considered to be stable, when symptoms are manageable and not rapidly progressive, and when there has not been recent infarction, procedural intervention or evidence of ongoing cardiac necrosis. Stable IHD confers a 10–13% annualized risk of major adverse cardiac events (MACE) [3].

Characterization of stable IHD is fundamental to appropriate diagnostic testing, risk stratification, prevention and medical or procedural intervention. Recent precision-based literature suggests that differences in lifestyles; exposure to varied natural, social and personal environments; and other unique social determinants of health can significantly alter the course of one's cardiovascular disease [4]. To effectively formulate such a personalized approach towards IHD, sex-based characteristics and stratified research should be incorporated [5]. However, to date, most cardiovascular research on sex-specific differences has focused primarily on variations in disease phenotypes and treatment response. While sex-based differences in IHD symptomatology [6] and vascular aging [7] are well known [6], sex-specific differences in remote patient monitoring have not been well studied.

The Prediction, Risk, and Evaluation of Major Adverse Cardiac Events (PRE–MACE) study monitored patients with stable IHD using remote patient biometric and psychometric data and levels of serum biomarkers predictive for MACE over 12 weeks. We sought to determine sex-based differences in demographics, adherence, biometric data, sleep, psychometric data, and biomarkers, among patients with stable IHD.

## Methods

The PRE–MACE study evaluated *N* = 198 patients for 12 weeks, as previously published [8]. In brief, patients were enrolled with stable IHD and intermediate risk of MACE, which corresponds to an annualized risk of MACE of 10–13%. Patients were recruited from four sources: pre-existing research registries of patients with clinically documented stable IHD, a large, academic hospital-based cardiac rehabilitation center, a tertiary care women’s heart center, and physician referral. Inclusion criteria were as follows: 18 years of age or older, current diagnosis or history of IHD, owning or having access to a smartphone or device, and the willingness to return for required follow-up visits. Patients were excluded if they had symptoms or signs of acute coronary syndrome and/or heart failure (Class III/Class IV), planned revascularization or valve surgery, any comorbidity precluding 12 month survival, or a history of psychiatric disorders or substance abuse. All participating patients provided signed informed consent, using forms and procedures in accordance with institutional guidelines and approved by the institutional review board.

Biometric data were collected from a wearable Fitbit Charge 2 (Fitbit Inc., San Francisco, CA, USA) device. The patients were instructed to wear the device on their wrist at all times, except when bathing, swimming or partaking in other activities involving water, or while the device was charging. Fitbit data was continuously synced and uploaded to the Fitabase (Small Steps Labs LLC., San Diego, CA, USA) system. Adherence to Fitbit data collection was defined as the percentage of study hour data entries that contained non-zero or non-null values.

Psychometric data were acquired using the Kansas City Cardiomyopathy Questionnaire (KCCQ), the Seattle Angina Questionnaire (SAQ), and the following short-form subscales of the Patient-Reported Outcomes Measurement Information System (PROMIS): depression, emotional distress/anxiety, fatigue, physical function, sleep disturbance, social isolation, global mental health, and global physical health. The responses to these questionnaires were collected through a mobile phone application or web browser using the HealthLoop electronic platform. Adherence to questionnaire data collection was defined as the mean percentage of weeks of questioning to which study patients responded, up to 12 weeks in the study. Data regarding adherence to study protocols and sleep duration were obtained as previously described [9].

Serum biomarkers, including u-hs-cTnI, an index of cardiovascular damage; NT-proBNP, an index of cardiovascular strain; and hs-CRP, an index of cardiovascular inflammation, were drawn at the baseline visit. Levels of each biomarker were considered elevated if u-hs-cTnI was greater than 5 ng/L, NT-pro-BNP was greater than 300 pgram/mL and hs-CRP was greater than 0.3 mg/dL, respectively.

Percent frequencies and means ± standard deviation (SD) were used to describe categorical and normally distributed continuous variables stratified by sex, respectively. For non-normally distributed continuous variables, medians and interquartile ranges were used and natural log transformation was performed, before inclusion in regression analyses. Statistical analysis included *t* tests for normally distributed continuous variables, Wilcoxon rank sum for nonparametric variables, and chi-square tests for categorical variables. A significance level of 0.05 was used for all tests. Regression analyses were performed using the stats package (v3.5.1) in R v1.1.453 (R foundation, Vienna, Austria). Multivariable regression analyses examined sex-based differences in adherence to study protocols, adjusting for comorbidities and differences in baseline characteristics, including age, body mass index (BMI), diastolic blood pressure, HDL cholesterol, triglycerides, NT-proBNP and u-hs-cTnI levels.

## Results

### Demographics

The mean age of the cohort was 65.5 ± 11 years (± SD), and 119 patients (60%) were females. Overall, 42 patients (21%) in the cohort had a history of diabetes mellitus and 77 patients (39%) had a history of tobacco use. In the context of medication use, 168 patients (85%) in the cohort were taking aspirin, 168 patients (85%) were taking a statin and 128 patients (65%) were taking a β‐blocker, on enrollment.

Compared with males, females had lower diastolic blood pressures (*p* = 0.019), higher HDL cholesterol (*p* < 0.001) and lower triglycerides (*p* = 0.002). There were no statistically significant differences observed in age, race, body mass index, systolic blood pressure, history of diabetes, history of smoking, total cholesterol, LDL cholesterol, aspirin use and statin use, between females and males (Table 1).

**Table 1:** Demographics and biometric, psychometric, sleep and biomarker data

	Females *N* = 119 (60%)	Males *N* = 79 (40%)	*P* value
**Demographics**	Mean ± SD or % or Median (IQR)		
Age, years	64 ± 11	67 ± 11	0.06
Non-white race	33	22	0.12
Body mass index, kg/m2	27.5 (23.8, 31.7)	26.6 (24.6, 29.9)	0.73
Systolic BP, mmHg	120 (110,132)	122 (112, 136)	0.16
Diastolic BP, mmHg	68 (62, 74)	70 (66, 76)	**0.019**
History of diabetes, %	19	23	0.68
History of smoking, %	37	41	0.73
Total cholesterol, mg/dL	148 (127, 190)	149 (123, 182)	0.79
LDL cholesterol, mg/dL	68 (53, 97)	77 (57, 106)	0.07
HDL cholesterol, mg/dL	54 (43, 67)	42 (36, 50)	** < 0.001**
Triglycerides, mg/dL	88 (64, 119)	110 (76, 158)	**0.002**
Aspirin use, %	82	89	0.32
Statin use, %	85	86	0.98
**Biometric indices**	Median (IQR) or Mean ± SD		
Steps per week	5,559 (3,423, 7,959)	6,008 (4,807, 9,080)	0.06
Physical activity, min/week			
Light level	202 (146, 251)	180 (156, 227)	0.38
Moderate/vigorous level	39 (32, 53)	37 (24, 51)	0.24
Heart rate, beats/min			
During rest	67 ± 8	66 ± 8	0.39
During activity	84 ± 11	82 ± 8	0.24
Psychometric indices*	Mean ± SD or Median (IQR)		
PROMIS scores			
Global physical health	45 ± 9	50 ± 9	**0.001**
Physical function	43 (38, 51)	49 (43, 53)	**0.001**
Depression	52 (41, 56)	41 (41, 54)	**0.020**
Anxiety	54 (49, 58)	52 (44, 57)	**0.039**
Fatigue	53 (46, 61)	49 (40, 55)	**0.003**
Sleep disturbance	54 (46, 57)	48 (44, 53)	** < 0.001**
SAQ-7 scores			
Overall	72 (51, 85)	82 (73, 90)	**0.001**
Physical limitation	75 (50, 100)	92 (67, 100)	**0.013**
KCCQ scores			
Quality of life	63 (38, 88)	75 (63, 88)	** < 0.001**
Physical limitation	83 (52, 100)	92 (67, 100)	**0.025**
Sleep duration** (hours per day)	6.9 (6.3, 7.7)	6.8 (6.0, 7.3)	**0.029**
Cardiovascular risk biomarkers^†^	Median (IQR)		
NT-proBNP (pgram/ml)	104 (59, 207)	185 (75, 527)	**0.001**
u-hs-cTnI (ng/L)	1.25 (0.79, 2.68)	4.50 (1.97, 7.12)	** < 0.001**
hs-CRP (mg/dL)	0.15 (0.06, 0.40)	0.10 (0.04, 0.24)	0.08
Proportion with elevated biomarker levels^†^	%		
NT-proBNP	16.0% (19/119)	32.9% (26/79)	**0.005**
u-hs-cTnI	16.0% (19/119)	46.8% (37/79)	** < 0.001**
hs-CRP	31.9% (38/119)	21.5% (17/79)	0.11

*For the PROMIS, higher scores represented more of the concept being measured; for both the KCCQ and SAQ, the range of possible subscale scores was 0 to 100, with 100 representing the least burden of symptoms

**Values are based on entries that contained non-zero or non-NA values

^†^*NT‐proBNP* N‐terminal pro‐b‐type natriuretic peptide); *u‐hs‐cTnI* ultra‐high sensitivity cardiac‐specific troponin I), *hs‐CRP* high‐sensitivity C‐reactive protein; levels of each biomarker were considered elevated if u-hs-cTnI > 5 ng/L, NT-pro-BNP > 300 pgram/mL and hs-CRP > 0.3 mg/dL, respectively

Significant *p*-values are in bold

### Adherence

There was no difference in adherence to collection of Fitbit data and overall SAQ-7 questionnaire data between females and males. However, females were less adherent than males to the collection of the remaining psychometric data, including completion of the SAQ-7 physical limitations, KCCQ, and PROMIS questionnaires (*p* < 0.05, Table 2).

**Table 2:** Differences in adherence to fitbit and questionnaire data collection between males and females

	% Difference in adherence^†^	*P* value
FitBit* Median (IQR)	1.80 (− 0.5, 32.8)	0.49
Questionnaires** Mean (95% CI)		
PROMIS		
Global physical health	9.76 (1.27, 18.2)	**0.025**
Physical function	10.2 (1.71, 18.7)	**0.020**
Depression	10.01 (1.49, 18.5)	**0.023**
Anxiety	9.91 (1.38, 18.4)	**0.024**
Fatigue	9.90 (1.41, 18.4)	**0.023**
Sleep disturbance	9.79 (1.26, 18.3)	**0.026**
SAQ-7		
Overall	7.46 (− 1.18, 16.9)	0.09
Physical limitation	14.3(5.20, 23.3)	**0.002**
KCCQ		
Quality of life	9.14 (0.63, 17.7)	**0.037**
Physical limitation	12.3 (3.61, 20.9)	**0.006**

*Value is difference in percentage of hours that contained non-zero or non-NA values, between males and females

**Values are differences in mean percentage of weeks of questioning to which patients responded, up to 12 weeks in the study, between males and females. This model was adjusted for age, body mass index, diastolic blood pressure, HDL cholesterol, triglycerides, NT-proBNP and u-hs-cTnI levels

^†^Values represent % difference in adherence, calculated as males—females

Significant *p*-values are in bold

### Biometric physical activity and sleep

**Fig. 1:**
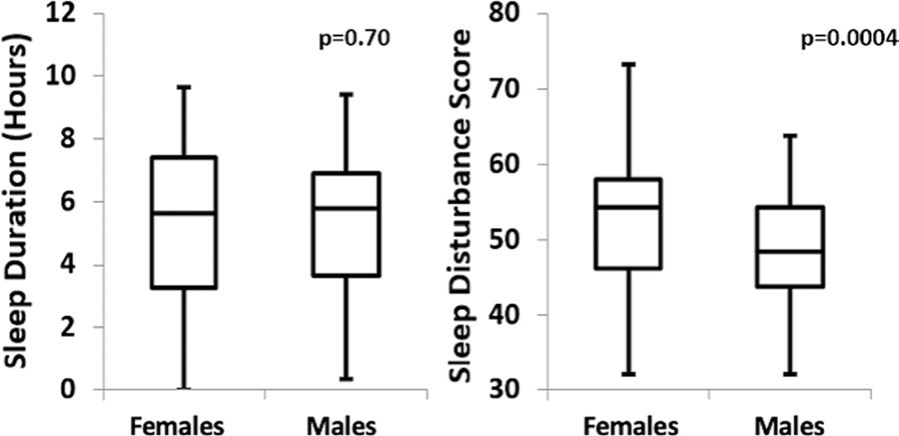
Fitbit sleep duration and PROMIS sleep disturbance score in Males vs. Females. The variation in hours of sleep duration (as measured by Fitbit) and perceived sleep quality (as measured by PROMIS questionnaire sleep disturbance score), between sexes

There were no statistically significant differences observed in minutes of physical activity per week, rigor of exercise, or heart rate, between females and males. While step count was higher in males, this difference was not statistically significant. There was a statistically significant difference in sleep duration between sexes, with females sleeping 6 min longer. (*p* = 0.029). However, females reported higher PROMIS sleep disturbance scores (*p* < 0.001). These relationships are shown in Fig. 1.

### Psychometric variables

Females reported significantly lower global physical health (*p* = 0.001) and physical function PROMIS scores (*p* = 0.001), in addition to significantly higher scores for depression (*p* = 0.02), anxiety (*p* = 0.039), fatigue (*p* = 0.003), and sleep disturbance (*p* < 0.001), compared with males. Females had significantly lower (worse) SAQ-7 overall (*p* = 0.001) and physical limitation scores (*p* = 0.013). KCCQ quality of life (*p* < 0.001) and physical limitations scores (*p* = 0.025) were also poorer among females (Table 1).

### Biomarkers

28% of patients had elevated u-hs-cTnI (> 5 ng/L), 23% had elevated NT-pro-BNP (> 300 pgram/mL) and 28% had elevated hs-CRP levels (> 0.3 mg/dL, Table 1). Males were found to have elevated NT-proBNP (*p* = 0.005) and u-hs-cTnI (*p* < 0.001) levels more often than females. However, elevated hs-CRP levels were more often present in females than males, although this difference was not statistically significant.

## Discussion

Our findings provide novel insight into sex-based differences in remote patient monitoring in patients with stable IHD. Females were less adherent to completion than males for the psychometric SAQ-7 physical limitations, KCCQ, and PROMIS questionnaires. There was no difference in the collection of biometric Fitbit data. Our findings of lower psychometric adherence in females may be due to a recently reported statistic by the Bureau of Labor Statistics which indicates that females work longer hours than males, in addition to fulfilling more other daily commitments at home [10]. Other studies have found that males are more adherent to cardiac rehabilitation than females [11], another activity requiring a significant investment of time. These findings further support that lifestyle factors may affect a females’ participation in both remote patient monitoring and certain medical interventions.

While this study reports sex-based differences in adherence to collection of psychometric data, other studies on sex-based differences with adherence to medical treatment have shown that males are less adherent than females to heart failure [12] and hypertension [13] treatment. Hence, awareness of the factors responsible for differences in adherence to data collection and treatment among females and males can allow for the development of IHD treatment plans and interventions that accommodate these sex-specific attitudes and lifestyles.

While females reported higher sleep disturbance than males on the PROMIS questionnaire, females had a measured median sleep duration that was 6 min longer than males. Our finding of higher reported sleep disturbance among females is consistent with prior literature, which found that poorer quality of sleep and fatigue was more often reported among females than males [14]. In addition, females in our study reported more fatigue, which could further be accounted for by less restorative sleep, despite having 6  more minutes of sleep. This supports the notion that sleep duration in the population is not clinically significant, as it does not translate to meaningful sleep with supporting research finding that females also self-report excessive sleep duration, or hypersomnia [14]. This discrepancy, between measured sleep duration and reported sleep disturbance, reiterates a possible discordance between actual and perceived health. This finding further highlights the concept that sleep quantity does not correspond with quality, particularly among females with IHD.

Females reported worse psychometric measures than males, including lower global physical health and physical function scores, as well as worse SAQ fatigue, sleep disturbance, and quality of life scores. Our findings are consistent with a previous study, which concluded that SAQ scores at the time of angiography, in addition to 1 year later, were significantly better in males when compared with females [15]. A similar analysis found that females were more likely to report poor health and disability in general, even following adjustment for demographic characteristics [16]. Thus, regardless of the mechanism underlying these differences, these findings suggest potential for development of sex-specific care models for stable IHD.

Despite females’ poorer self-reported psychometric measures, females and males did not have significant differences in biometric indices as measured by Fitbit, including minutes of physical activity per week, rigor or exercise and heart rate. The lack of a difference in reported rigor of exercise between sexes may be surprising, as studies have found that males tend to self-report higher physical activity than females [17]. The latter may reflect an underlying social desirability bias, whereby males avoid responses that could damage their self-image and/or reputation in the community. This is a perception which is associated with poorer clinical outcomes [18]. As the finding of higher step count among males approached significance, this relationship should be subsequently investigated in a larger cohort.

There were also sex-based differences in serum biomarker level elevations. Most individuals within our cohort with elevations in NT-proBNP and u-hs-cTnI, markers of cardiovascular strain and damage, respectively, were males. Our findings are consistent with those of recent studies, which have identified elevated levels of these biomarkers among males [19, 20]. The observed difference in u-hs-cTnI levels in this study may specifically reflect sex-based variations in body composition, cardiac mass as well as mechanisms of cardiomyocyte apoptosis in the setting of cardiac remodeling [19].

Our previous PRE–MACE study revealed that at baseline, elevated NT-proBNP levels were associated with poorer psychometric and biometric indices [21]. However, in this study, despite more commonly having elevated NT-proBNP levels, males had better psychometric and similar biometric data, when compared with females. This variation likely reflects the heterogeneity of factors contributory to NT-proBNP levels, extending beyond biometric and psychometric indices and sex.

These findings highlight multiple important sex-based differences in remote patient monitoring in the stable IHD population. However, there were some limitations. Adherence may have been increased by subject awareness of being monitored and already owning a smart device or computer. Our patients were also recruited from a cardiac rehabilitation program at a tertiary women’s heart center and demonstrated a high medication compliance rate [8]. Thus, these results may not be reflective of a more general IHD population. It is also important to consider that given the average age of the cohort was greater than 65 years, the majority of the female patients were in menopause. However, as only two were on transdermal hormone replacement therapy, we do not feel this significantly affected our findings. In terms of sleep, the accuracy of the observed difference in sleep duration between sexes may have been influenced by decreased adherence to sleep data collection masking the actual number of hours slept. Furthermore, we previously postulated that the sleep estimate provided by Fitbit may underestimate the total sleep for certain patients, due to overnight removal of the device resulting in non-wear time [9].

## Perspectives and significance

Our study raises important findings regarding sex-specific perceptions of health and adherence to remote patient monitoring practices. The observed discrepancies between biometric and psychometric data and the variations in serum biomarker level elevations seen among females and males, support the need to develop and study sex-specific counseling and therapeutic interventions in larger cohorts within the IHD population.

## Conclusions

There are sex-based differences in remotely monitored psychometric measures, biometric sleep, and biomarker data, which may improve sex-specific understanding of IHD. Females report poorer psychometric and sleep quality scores than males. Elevated levels of biomarkers for MACE are more common in males. Females are less adherent to collection of psychometric data. Sex-based differences in remote patient monitoring measures support the benefit of sex-specific data gathering and care delivery models. However, before we can develop this sex-specific care, we must account for sex-based differences in adherence to remote monitoring protocols, which could result in incomplete and potentially inaccurate attempts at data collection via these novel methods. Indeed, as in other areas of engineering, knowing our users is fundamental to the success of any remote monitoring platform. Further prospective investigation with a larger cohort could evaluate the benefits of a tailored, precision medicine-based approach towards optimization of adherence to remote data collection, diagnosis and therapy, based upon these sex-based differences in attitude, perception and MACE biomarkers.

## Data Availability

All data generated or analyzed during this study are included in the published article.
